# History and Current Status of Development and Use of Viral Insecticides in China

**DOI:** 10.3390/v7010306

**Published:** 2015-01-20

**Authors:** Xiulian Sun

**Affiliations:** Key Laboratory of Agricultural and Environmental Microbiology, Wuhan Institute of Virology, Chinese Academy of Sciences, Wuhan 430071, China; E-Mail: sunxl@wh.iov.cn; Tel.: +86-27-8719-8641; Fax: +86-27-8719-8072

**Keywords:** viral insecticides, commercialization, genetic modification

## Abstract

The use of insect viruses as biological control agents started in the early 1960s in China. To date, more than 32 viruses have been used to control insect pests in agriculture, forestry, pastures, and domestic gardens in China. In 2014, 57 products from 11 viruses were authorized as commercial viral insecticides by the Ministry of Agriculture of China. Approximately 1600 tons of viral insecticidal formulations have been produced annually in recent years, accounting for about 0.2% of the total insecticide output of China. The development and use of *Helicoverpa armigera* nucleopolyhedrovirus, *Mamestra brassicae* nucleopolyhedrovirus, *Spodoptera litura* nucleopolyhedrovirus, and *Periplaneta fuliginosa* densovirus are discussed as case studies. Additionally, some baculoviruses have been genetically modified to improve their killing rate, infectivity, and ultraviolet resistance. In this context, the biosafety assessment of a genetically modified *Helicoverpa armigera* nucleopolyhedrovirus is discussed.

## 1. Introduction

Research on insect viruses in China started with the *Bombyx mori* nucleopolyhedrovirus in the mid-1950s [1] and, to date, more than 200 insect virus isolates have been recorded in China. Viruses from several families, such as *Baculoviridae*, *Reoviridae*, *Densoviridae*, and *Entomopoxvirinae* can cause epizootics in natural populations of insects. Therefore, such viruses are attractive biological agents for the control of insect pests in agriculture, forestry, pasture, and domestic gardens. Here, the history and current status of the development and use of viral insecticides in China over the past 50 years are reviewed.

## 2. Development and Commercialization of Wild-Type Viruses as Bioinsecticides

The earliest experiments investigating the use of insect viruses as biological control agents in China started in the early 1960s, e.g., two baculoviruses were inoculated into their noctuid hosts, *Agrotis segetum* and *Apamea sordens* [2]. These experiments were followed by in-depth field efficacy studies where the *A. segetum* granulovirus (AgseGV) was used to infect its *A. segetum* host and to test its biosafety in silkworms, mice, and rabbits [3]. The studies included formulation tests [4] and virus production and application [5]. Since then, more than 32 insect viruses have been investigated as potential bioinsecticides (Table 1).

**Figure 1:**
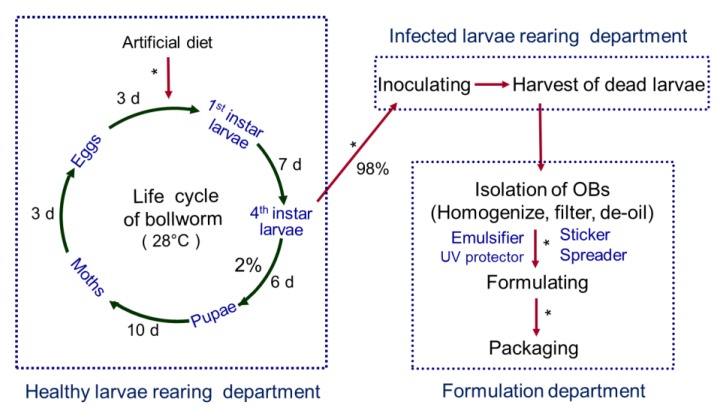
Schematic representation of the production of HearNPV insecticide. The steps with an asterisk (*) can be implemented by robotics.

Of the heliothine insects, *Helicoverpa armigera* causes the most serious damage to agricultural crops. Its larval stage attacks a wide variety of agricultural crops including cotton, pepper, tomato, tobacco, maize, sorghum, sunflower, pigeon pea, chickpea, groundnut, soybean, and okra. In 1975, a *Helicoverpa armigera* nucleopolyhedrovirus (HearNPV) was isolated from diseased *H. armigera* larvae in Hubei Province of China [6]. It has been subsequently subjected to toxological and pathogenicity tests on vertebrates [7]. HearNPV has been mass produced as a viral pesticide via continuous rearing of *H. armigera* on an artificial diet (Figure 1, modified from [8]). HearNPV emulsifiable suspension was authorized as the first commercialized viral insecticide in 1993 by the Institute for the Control of Agrochemicals, the Ministry of Agriculture of China (ICAMA) [9]. In 2014, there were 17 products that included HearNPV as the main insecticidal component and these products were made by 10 different companies (Table 2). The products were formulated as emulsifiable concentrates (ECs) containing 2 × 10^9^ occlusion bodies (OBs)/mL, wettable powders (WPs) containing 2 × 10^9^ OBs/g, or water-dispersible granules (WDG) containing 6 × 10^10^ OBs/g (ICAMA, 2014). One product contained HearNPV at 1 × 10^9^ OBs/g and 16% phoxim, an organophosphate insecticide with low toxicity to experimental animals. For bollworm control, the products were used at 1.2–2.25 × 10^12^ OBs/ha as a foliar spray for cotton, pepper, or tobacco plants. According to a questionnaire survey of insecticide producers conducted by ICAMA, total production of HearNPV formulations was 968 tons in 2012 [10], making it the most produced viral insecticide in China (Table 2).

**Table 1:** Insect viruses evaluated as potential bioinsecticides in China.

No.	Virus Name	Target Insect (s)	Host Crops	References
1	*Agrotis segetum* GV	*A. segetum*	Maize, beet, tomato, tobacco, cotton, cabbage	[2,3,4,5]
2	*Andraca bipunctata* GV	*A. bipunctata*	Tea	[11,12,13]
3	*Apocheima cinerarius* NPV	*A. cinerarius*	Poplar	[14,15,16]
4	*Aporia crataegi* NPV	*A. crataegi*	Hawthorn, apple	[17,18,19]
5	*Autographa californica* NPV	*S. exigua*, *Hellulo undalis*, *Pectinophora gossypiella*	Cabbage, cotton	[20,21]
6	*Buzura suppressaria* NPV	*B. suppressaria*	Tea, metasequoia	[22,23,24]
7	*Clostera anastomosis* GV	*C. anastomosis*	Poplar, willow	[25,26,27]
8	*Clostera anachoreta* GV	*C. anachoreta*	Poplar	[28,29]
9	*Cydia pomonella* GV	*C. pomonella*	Apple	[30,31,32]
10	*Dendrolimus punctatus* CPV	*D. punctatus*, *D. kikuchii*, *D. spectabilis*, *D. supreans*	Pine	[33,34,35,36]
11	*Ectropis oblique* NPV	*E. obliqua*	Tea	[37,38,39,40]
12	*Ectropis grisescens* NPV	*E. grisescens*	Tea	[41,42,43]
13	*Eranhis ankeraria* NPV	*E. ankeraria*	Larch	[44,45]
14	*Euproctis pseudoconspersa* NPV	*E. pseudoconspersa*	Tea	[46,47,48]
15	*Euproctis similis* NPV	*E. similis*	Mulberry	[49,50]
16	*Gynaephora ruoergensis* NPV	*G. ruoergensis*	Grass	[51,52,53,54]
17	*Helicoverpa armigera* NPV	*H. armigera*, *Heliothis assulta*	Cotton, pepper, tobacco	[6,7,8]
18	*Hyphantria cunea* NPV	*H. cunea*	Ash, heaven tree, chinar	[55,56,57,58]
19	*Lymantria dispar* NPV	*L. dispar*	Oak, larch, birch	[59,60,61]
20	*Lymantria xylina* NPV	*L. xylina*	Coast oak	[62,63,64,65]
21	*Iragoides fasciata* NPV	*I. fasciata*	Tea	[66,67,68]
22	*Mamestra brassicae* NPV	*M. brassicae*, *H. armigera*, *S. exigua*	Cabbage, cowpea	[69,70]
23	*Mythimna separata* NPV	*M. separata*	Wheat, rice, corn	[71,72,73]
24	*Parocneria orienta* NPV	*P. orienta*	Cypress	[74,75,76]
25	*Periplaneta fuliginosa* DNV	*P. fuliginosa*	House	[77,78]
26	*Pieris brassicae* GV	*P. brassicae*	Cabbage	[79,80]
27	*Pieris rapae* GV	*P. rapae*	Cabbage, broccoli	[81,82,83]
28	*Plusia agnate* NPV	*P. agnate*	Soybean, rape, broccoli	[84,85,86]
29	*Plutella Xylostella* GV	*P. xylostella*	Radish, cabbage, mustard	[87,88]
30	*Spodoptera exigua* NPV	*S. exigua*	Beet, cabbage	[89,90,91]
31	*Spodoptera litura* NPV	*S. litura*	Cabbage, tobacco	[92,93,94,95]
32	*Sucra jujube* NPV	*S. jujuba*	Jujube	[96,97,98,99]

**Table 2:** Viruses authorized by the Ministry of Agriculture of China as commercial insecticide.

Virus Name	Target Insect	Crops	No. of Producers [9]	No. of Products [9]	Production in 2012 (tons) [10]
*Helicoverpa armigera* NPV	Cotton bollworm	Cotton	10	17	968
*Mamestra brassicae* NPV	Cabbage moth	Vegetables	1	1	220
*Autographa californica* NPV	Beet armyworm	Vegetables	3	3	175
*Spodoptera exigua* NPV	Beet armyworm	Vegetables	3	4	70
*Spodoptera litura* NPV	Cotton leafworm	Vegetables	2	2	53
*Plutella xylostella* GV	Diamondback moth	Vegetables	1	1	11
*Dendrolimus punctatus* CPV	Masson pine moth	Pine	2	4	6
*Ectropis oblique* NPV	Tea geometrid	Tea	1	1	-
*Euproctis pseudoconspersa* NPV	Tea caterpillar	Tea	1	1	-
*Pieris rapae* GV	Cabbage white butterfly	Vegetables	1	1	-
*Periplaneta fuliginosa* DNV	Cockroach	Sanitation	1	1	-

*Spodoptera litura* (Fab.) is a serious pest to vegetables such as broccoli, beans, cabbage, and dasheen in Southern China. Following its discovery in Guangzhou Province [92], *Spodoptera litura* nucleopolyhedrovirus (SpliNPV) has been studied extensively to determine its infectivity levels [93], mass production potential [94], and biosafety towards bees, fish, silkworm, mice, rabbits, and monkeys [95]. Since 1997, SpliNPV has been produced as a commercialized insecticide via continuous rearing of the host larvae on an artificial diet [94]. The products were formulated as ECs at 1 × 10^9^ OBs/g or WDGs at 2 × 10^10^ OBs/g. The formulations were applied to vegetables at 6.0 × 10^11^–1.2 × 10^12^ OBs/ha to control leafworm. Production of SpliNPV formulations was 53 tons in 2012 [10].

**Figure 2:**
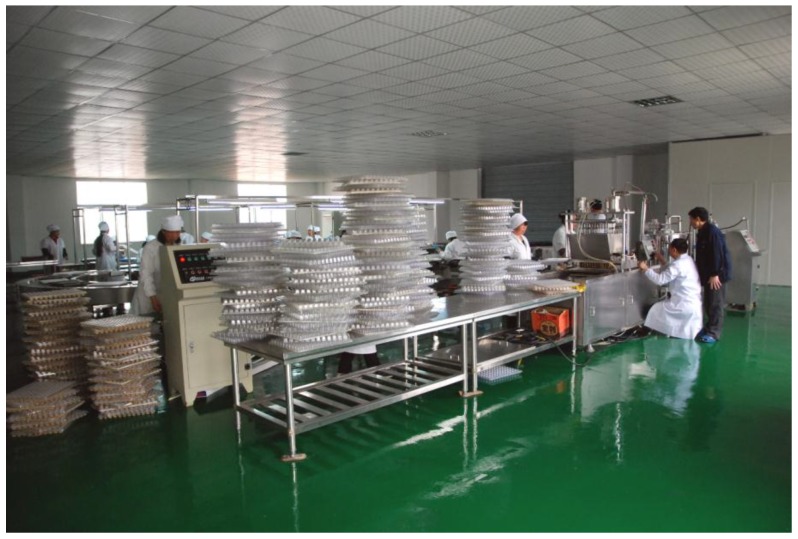
A diet distributing line for rearing *H. armigera* larvae established in Yichun, Jiangxi Province, China.

*Mamestra brassicae* nucleopolyhedrovirus (MabrNPV) has a wide host range, including 32 species spanning five Lepidoptera families [100]. Some of its targets are important pests, such as *Plutella xylostella*, *H. armigera*, *S. exigua*, and *Xestia c-nigrum*. A MabrNPV, originally isolated from *M. brassicae* larval cadavers on oilseed rape in Tai’an, Shandong in 1979 [69], was successfully developed as an insecticide produced in either *H. armigera* or *S. exigua* reared on an artificial diet [70]. The product, formulated as a WP at 2 × 10^10^ OBs/g, met the EU standard 889/2008 and was certified as an organic product by ECOCERT INPUTS (France) in 2013. It was applied to vegetables to control various insect pests at 2.7 × 10^12^–3.6 × 10^12^ OBs/ha. A MabrNPV production line with a 2000-ton formulation capacity was built in Yichun, Jiangxi Province, China (Figure 2) and enabled production of 220 tons of MabrNPV formulations in 2012 [10] and 400 tons in 2013 [101].

Two other NPVs that have also undergone large-scale production are *Autographa californica* multiple nucleopolyhedrovirus (AcMNPV) and *Spodoptera* *exigua* MNPV (SeMNPV); both of these were used to control vegetable-eating insect pests in China on a large scale. In 2012, production of AcMNPV and SeMNPV was 175 and 70 tons, respectively [10].

Cockroach infestation is a serious problem in most urban areas of China. The smoky brown cockroach, *Periplaneta fuliginosa* (Serville), is an increasingly important peridomestic pest throughout much of the Southeastern United States, Japan, and Southeast Asia. *Periplaneta fuliginosa* densovirus (PefuDNV), purified from diseased smoky-brown cockroaches in China [77], has been commercially produceed since 2008 [78]. The product was formulated in combination with a sex pheromone as a paste containing 6 × 10^3^ particles/g. The paste was used in commercially important areas where the cockroaches were found at high frequency. In 2013, a total of 5500 kg of PefuDNV paste was produced, which treats an area of about 5.5 million m^2^ [102].

## 3. Development of Recombinant Viruses as Bioinsecticides

Despite several cases of successful viral insecticide use in China, insecticide failures were also frequently documented. The main problems related to their relatively slow speed of action, low virulence against older insect instars, and ultraviolet radiation sensitivity. Subsequently, genetic techniques were adopted to overcome such issues. For example, genetic modification was used to improve the efficacy of HearNPV by inserting an insect-specific scorpion toxin (AaIT) gene, resulting in the recombinant virus, HearNPV-AaIT [103]. Later on, HearNPV was similarly modified by inserting the basement membrane-degrading protease into its genome [104]. Application of HearNPV-AaIT on cotton protected the fruit from damage by the bollworm better than the application of wild-type HearNPV over the cotton seasons of 2000 and 2001. Indeed, the yield of cotton lint from HearNPV-AaIT-treated plants was about 22% higher than that from plantations treated with the wild-type virus alone in 2000 and 2001 [103].

To assess the risk of releasing recombinant HearNPV into the environment, the following were investigated: the effect of HearNPV-AaIT on non-target species, the possibility of AaIT gene flow to other organisms, and the environmental fitness of HearNPV-AaIT. HearNPV-AaIT was non-toxic to rats, with a median lethal dose >2 g/kg against female and male rats administered either intradermally or orally. Furthermore, there were no pathological responses when rats were inoculated with the recombinant virus, and followed by bone marrow polychromatic erythrocyte micronucleus test [105]. The recombinant virus was also found to be non-toxic to bobwhite quail, zebra fish, silkworm, and bees by oral administration. It was not anaphylactic to guinea pigs by percutaneous administration [105]. The AaIT toxin expressed in yeast was biologically active against *S. exigua* and *Argyrogramma agnata* larvae when injected into the hemocoele, whereas it was non-toxic to the two insects when it was administered orally [106], implying that the AaIT toxin expressed in *H. armigera* larvae infected by HearNPV-AaIT are safe to the predatory insects. The possibility that the AaIT gene from recombinant HearNPV might be transferred to other organisms, such as cotton-pathogenic fungus (*Verticillium dahliae*), ladybeetles (*Propylaea japonica*), and aphids (*Rhopalosiphum pseudobrassicae*), was very low when detected by polymerase chain reaction and dot-blot hybridization [107]. Based on these results, there was no evidence that this recombinant baculovirus posed an increased hazard to non-target organisms or had a deleterious effect on the environment when compared with wild-type viruses. As part of the registration process for commercial use of HearNPV-AaIT in China, a safety data package for the virus has been submitted to the Ministry of Agriculture, P.R. China.

In addition to baculovirus killing rates, the degree of infectivity and ultraviolet resistance of these viruses are also important factors affecting their performance in the field [108]. A recombinant HearNPV expressing GP64 from AcMNPV had been constructed. Bioassays showed that the LC_50_ (median lethal concentration) of this recombinant virus reduced to 20% of that of the control virus [109]. In terms of ultraviolet resistance, improvements have been made by constructing AcMNPV recombinants displaying nano-material binding peptides [110], and [Jin Li, unpublished data]. These constructs have the potential to improve baculovirus insecticides for future use in the field.

## 4. Perspectives on Use of Insect Viruses for Pest Control in China

In 2012, about 1600 tons of viral insecticidal formulations were produced, accounting for about 0.2% of the total insecticide output in China (derived from [10]). Currently, increasing demand for healthy food and environmentally friendly pesticides drives the market for biopesticide production. Improved viral products, a program of farmer education, prohibitively low acceptable chemical pesticide residues and robust government policies on viral pesticide use were important in enabling large-scale uptake of viral insecticides. To date, 34 government standards for chemical pesticidal residues on agricultural and food products have been employed in China. In recent years, ICAMA adopted a series of favorable policies on the registration process of biopesticides, e.g., remission of the request of pesticide residue data. Meanwhile, ICAMA also issued a serial of regulations to prohibit re-registration of high-toxic chemical pesticides. According to the ICAMA questionnaire survey in 2012, 67% of biopesticide producers planned to increase their input into the research and development of biopesticides, including viruses [10]. Since 2014, the Central Agricultural Broadcasting and Television School has organized a series of courses to teach farmers and agricultural technicians how to correctly use bio-insecticides. In 2012, the State Council of China issued a bio-industry development project, which indicated that government at all levels would give allowance on biopesticides producers and establish relative regulations to ensure the development of biopesticides [111]. Some local governments, such as that in Shanghai, also gave an allowance to farmers who applied biopesticides. The farmers received the allowance via dealers sustained by government agencies. These measures promote the future use of insect viruses in China.

## 5. Conclusions

During past 50 years, more than 32 viruses had been developed and used as insecticides in China and 11 viral insecticides had been successfully commercialized. As demands for the healthy food and environmental protection increase, it could be predicted more and intensiver viral pesticides are used in China in future.
